# A Genetic Variant in *pre-miR-27a* Is Associated with a Reduced Renal Cell Cancer Risk in a Chinese Population

**DOI:** 10.1371/journal.pone.0046566

**Published:** 2012-10-30

**Authors:** Danni Shi, Pu Li, Lan Ma, Dongyan Zhong, Haiyan Chu, Fu Yan, Qiang Lv, Chao Qin, Wei Wang, Meilin Wang, Na Tong, Zhengdong Zhang, Changjun Yin

**Affiliations:** 1 State Key Laboratory of Reproductive Medicine, Institute of Toxicology, Nanjing Medical University, Nanjing, China; 2 Department of Molecular & Genetic Toxicology, the Key Laboratory of Modern Toxicology of Ministry of Education, School of Public Health, Nanjing Medical University, Nanjing, China; 3 Department of Occupational Medicine and Environmental Health, Jiangsu Key Lab of Cancer Biomarkers, Prevention and Treatment, Cancer Center, Nanjing Medical University, Nanjing, China; 4 Department of Urology, the First Affiliated Hospital of Nanjing Medical University, Nanjing, China; IPO, Inst Port Oncology, Portugal

## Abstract

**Background:**

MicroRNAs (miRNAs) are a class of small non-coding RNAs to regulate cell differentiation, proliferation, development, and apoptosis. The single nucleotide polymorphism (SNP) rs895819 is located at the terminal loop of *pre-miR-27a*. Here, we aimed to investigate whether SNP rs895819 was associated with the development of renal cell cancer (RCC) in a Chinese population.

**Methods:**

In this case-control study, we recruited 594 RCC patients and 600 cancer-free controls with frequency matched by age and sex. We genotyped this polymorphism using the TaqMan assay and assessed the effect of this polymorphism on RCC survival. Logistic regression model was used to assess the genetic effects on the development of RCC and interactions between rs895819 polymorphism and risk factors.

**Results:**

Compared with AA homozygote, individuals carrying AG/GG genotypes had a statistically significant reduced susceptibility to RCC (adjusted OR = 0.71, 95% CI = 0.56–0.90). Furthermore, AG/GG genotypes were associated with reduced RCC susceptibility in localized clinical stage (adjusted OR = 0.71, 95% CI = 0.55–0.91), and similar effects were observed in well differentiated and poorly differentiated RCC (adjusted OR = 0.71, 95% CI = 0.55–0.93 for well differentiated, adjusted OR = 0.51, 95% CI = 0.28–0.93 for poorly differentiated). We also observed that rs895819 had multiplicative interactions with age and hypertension. However, the polymorphism did not influence the survival of RCC.

**Conclusion:**

Our results suggest that the *pre-miR-27a* rs895819 polymorphism can predict RCC risk in a Chinese population. Larger population-based prospective studies should be used to validate our findings.

## Introduction

Renal cell cancer (RCC) accounts for more than 90% of kidney carcinomas, and clear-cell renal carcinoma is the most common type in RCC [1], [2]. The incidences of RCC vary substantially worldwide, with higher rates in Europe and North America and lower rates in Asia and South America [1]. Rates among females are generally about half of those among males [1]. Though few risk factors are established for RCC, there are a number of predisposing conditions which are known to be related to the development of RCC, such as cigarette smoking, obesity, hypertension, diabetes, family history of cancer, and others [3], [4], [5]. However, only a part of the individuals exposed to these risk factors will develop RCC in their life time, suggesting that individual differences including genetic susceptibility factors may be one of the most critical agents in renal cell carcinogenesis.

MicroRNAs (miRNAs) are a class of endogenous, small and non-coding RNAs (∼22 nt), which are initially transcribed from genomic DNA to long primary transcripts (pri-miRNAs) and then are cleaved by nuclear Drosha into 60–70 nt hairpin-shaped precursor RNAs (pre-miRNAs) [6], [7]. Pre-miRNAs are exported to the cytoplasm by Exportin-5 and are further processed into ∼22 nt mature miRNA duplexes by the cleavage of Dicer [8], [9]. In association with RNA-induced silencing complex (RISC), miRNAs can induce mRNA degradation or translational repression by binding to the 3′-untranslated region of their target genes at the posttranscriptional level [10]. To date, it has been estimated that miRNAs modulate the expression of approximately 30% of human genes [11]. MiRNAs are involved in a wide range of biological processes including cell cycle regulation, apoptosis and stem cell maintenance, development, metabolism and aging [11]. It has been shown that miRNAs participate in human carcinogenesis as either tumor suppressors or oncogenes [12], [13]. Accumulative studies have suggested that single nucleotide polymorphisms (SNPs) or mutations could make a significant contribution to disease susceptibility and outcome. Genetic variants or mutations in miRNAs or pre-miRNAs may alter miRNA expression and/or maturation [14], [15].

One study has systematically identified 323 SNP in 227 known human miRNAs, and 12 SNPs are located within the miRNA precursors [14]. The SNP rs895819 is located at the loop of *pre-miR-27a* and involves an A>G nucleotide transition. Sun *et al.* [16] reported the polymorphism could lead to process variation, higher expression of miR-27a and eventually predisposition of gastric cancer [16]. While Yang *et al.* [17] found that G allele of rs895819 might impair the maturation of the miR-27a, thus, was associated with reduced familial breast cancer risk. Moreover, Mertens-Talcott *et al.* reported that in breast cancer cells, transfection of antisense miR-27a lead to increased expression of Zinc finger and BTB domain containing 10 (ZBTB10)( a putative Sp repressor) and these responses were accompanied by decreased expression of Sp-dependent survival and angiogenic genes, including *survivin*, vascular endothelial growth factor (*VEGF*), and VEGF receptor 1 (*VEGFR1*) [18]. However, over-expression of survivin was frequently observed in different types of cancer, including RCC [19]. To date, there is no study on the association between *pre-miR-27a* polymorphism and RCC susceptibility.

Based on our knowledge regarding the new polymorphism and biological function of *miR-27a*, we hypothesized that the *pre-miR-27a* polymorphism was associated with RCC susceptibility. To test this hypothesis, we genotyped this particular SNP rs895819 and assessed the association with risk of RCC in our ongoing case–control study in a Chinese population.

## Materials and Methods

### Study subjects

This study comprised 594 patients and 600 cancer-free controls. All subjects in our study are ethnic Han Chinese with no genetic relationship. All the patients were newly diagnosed with histopathologically confirmed incident RCC. Those cases that received chemotherapy or radio-therapy before surgery or had other type of cancer were excluded from the present study. Consecutive RCC patients were recruited between May 2004 and August 2010 at The First Affiliated Hospital of Nanjing Medical University, Nanjing, China. Disease was classified according to World Health Organization criteria and staged according to the American Joint Committee on Cancer TNM classification. The Fuhrman scale was used to assess tumor nuclear grade [20]. The controls were recruited from those who were seeking health care in the outpatient departments at the same hospital. The cancer-free controls were frequency matched by sex and age (±5 years) to the cases without individual history of cancer and family unrelated to the cases. A guided questionnaire on demographic and lifestyle factors was administered through face-to-face interviews by trained interviewers. Each patient donated 5 ml blood for genomic DNA extraction after a written informed consent obtaining from all subjects. This study was approved by the institutional review board of Nanjing Medical University.

For the survival analysis, 296 RCC cases enrolled in our ongoing cohort study from May 2004 to October 2009 were used. The patients were followed up prospectively every 6 months from the date receiving a confirmed diagnosis until death or last time of follow-up. The maximum follow-up time was 72 months (last follow-up in August 2010) and the median follow-up time was 19.3 months.

### Genotyping

**Figure 1:**
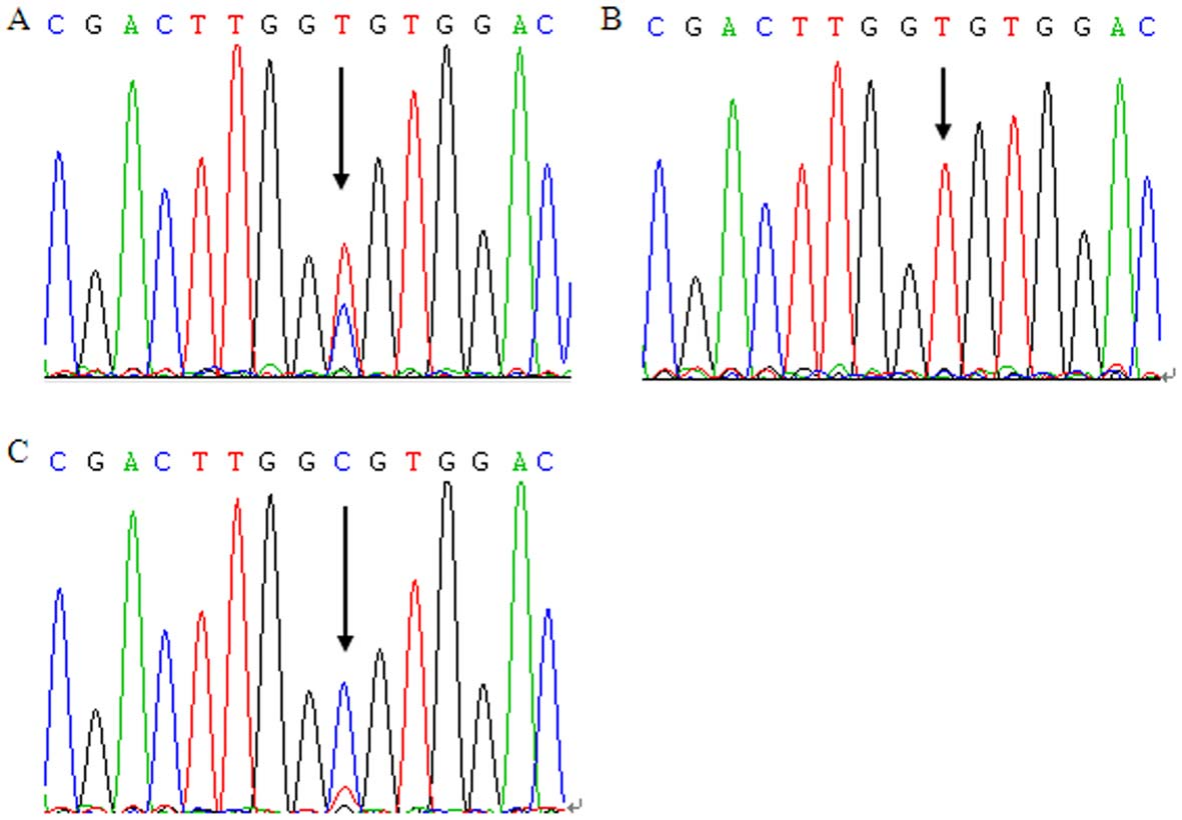
DNA sequencing chromatograms of three different samples of PCR products confirmed rs895819 polymorphism. (A) Double peaks labeled with an arrow represented the heterozygous genotype TC (AG). (B) Single peak labeled with an arrow represented the homozygous genotype TT (AA). (C) Single peak labeled with an arrow represented the homozygous genotype CC (GG).

Genomic DNA was isolated from leukocytes of venous blood by proteinase K digestion and phenol/chloroform extraction. Genotyping was performed with the TaqMan SNP Genotyping Assay using the 384-well 7900HT real time PCR System and the SDS 2.3 software for allelic discrimination (Applied Biosystems, Foster City, CA). The sequence of primers and probes are summarized in Table S1. Genomic DNA of 50 ng and 0.5×mix (TaKaRa Bio, JPN) was used for each reaction and amplification was performed under the following conditions: 50°C for 2 min, 95°C for 10 min followed by 45 cycles of 95°C for 15 sec, and 60°C for 1 min. The polymorphism analysis was performed by two persons independently in a blinded manner. About 10% of the samples were randomly selected for repeated genotyping for validation, and the results were 100% concordant. The PCR products of the SNP with different genotypes were selected and verified by direct sequencing (Figure 1).

### Analysis of hsa-miR-27a expression

To further evaluate correlation between hsa-miR-27a expression and rs895819 polymorphism *in vivo*, 25 surgically removed normal renal tissue samples containing 100% normal cells adjacent to tumors from patients of different genotypes were collected. Total RNA was extracted from these samples using Trizol Reagent (Invitrogen, Carlsbad, CA, U.S.) according to the manufacturer's protocol. Applied Biosystems 7900HT Real Time PCR System was used to perform real-time quantification PCR (Foster City, CA, U.S.) based on the SYBR-Green method (TaKaRa Bio, JPN). The primers used for amplification of hsa-miR-27a mRNA were 5′- ACACTCCAGCTGGGTTCACAGTGGCTAAG-3′ (forward) and 5′- TGGTGTCGTGGAGTCG-3′ (reverse), and the primers for U6 were 5′- CTCGCTTCGGCAGCACA-3′ (forward) and 5′- AACGCTTCACGAATTTGCGT-3′ (reverse). All reactions were conducted in triplicate. Fold changes were normalized to the expression levels of U6.

### Statistical analysis

Differences in the distributions of selected demographic variables and frequencies of genotypes between the cases and controls were evaluated by using the Student's *t*-test (for continuous variables) or Pearson's χ^2^-test (for categorical variables). Hardy-Weinberg equilibrium (HWE) of the controls' genotype frequencies was assessed by a goodness-of-fit χ^2^ test. The association between the SNP rs895819 polymorphism and RCC risk were estimated by computing odds ratios (ORs) and their 95% confidence intervals (CIs) from unconditional logistic regression analysis with the adjustment for possible confounders. The Kaplan-Meier method, log-rank test, univariate and multivariate Cox regression analyses were used to evaluate the effects of *pre-miR-27a* genotypes on the overall survival of patients with RCC. *P*<0.05 was considered statistically significant. All the statistical analyses were done with Statistical Analysis System software (9.1.3; SAS Institute, Cary, NC, U.S.) with two-sided *P* values. The statistical power was calculated by using the PS software (http://biostat.mc.vanderbilt.edu/twiki/bin/view/Main/PowerSampleSize).

## Results

### Characteristics of the study population

 The frequency distributions of selected demographic characteristics of the cases and controls are shown in Table 1. There were no differences between the patients and controls on age (*P* = 0.642), body mass index (BMI) (*P* = 0.074), sex (*P* = 0.222), smoking status (*P* = 0.127), pack-years of smoking (*P* = 0.251) and drinking status (*P* = 0.714). However, there were more hypertension patients (36.4%), and diabetics (13.5%) among the cases than among the controls (all *P*<0.05). The majority of patients (84.0%) had conventional clear cell carcinoma. Other patients who had papillary carcinoma, chromophobe carcinoma and unclassified were counted ninety-five (16%). Approximately 63.5% of patients were in stage I, 18.4%, 6.7%, and 11.4% was found to be in stage II, III, and IV, respectively. In addition, the frequencies of nuclear grades from I to IV were 18.2%, 48.0%, 24.7%, and 9.1%, respectively.

**Table 1:** Distribution of selected variables between the RCC cases and control subjects.

Variables	Cases (*n* = 594)		Controls (*n* = 600)		*P* a
	*n*	%	*n*	%	
Age (years) (mean ± SD)	56.8±11.9		57.8±11.3		0.143
≤57	305	51.4	300	50.0	0.642
>57	289	48.7	300	50.0	
BMI (kg/m^2^) (mean ± SD)	24.0±2.8		23.8±3.0		0.094
<24	292	49.2	326	54.3	0.074
≥24	302	50.8	274	45.7	
Sex					
Male	378	63.6	402	67.0	0.222
Female	216	36.4	198	33.0	
Smoking status					
Never	375	63.1	404	67.3	0.127
Ever	219	36.9	196	32.7	
Pack-years of smoking					
0	375	63.1	406	67.7	0.251
0–20	157	26.4	141	23.5	
>20	62	10.4	53	8.8	
Drinking status					
Never	427	71.9	437	72.8	0.714
Ever	167	28.1	163	27.2	
Hypertension					
No	378	63.6	433	72.2	0.002
Yes	216	36.4	167	27.8	
Diabetes					
No	514	86.5	565	94.2	<0.001
Yes	80	13.5	35	5.8	
Stage					
I	377	63.5			
II	109	18.4			
III	40	6.7			
IV	68	11.4			
Grade					
I	108	18.2			
II	285	48.0			
III	147	24.7			
IV	54	9.1			
Histology					
Clear cell	499	84.0			
Papillary	18	3.0			
Chromophobe	35	5.9			
Others	42	7.1			

a Student's t-test for age and BMI distributions between cases and controls; two-sided χ^2^ test for other selected variables between cases and controls.

SD, standard deviation; BMI, body mass index.

### Association between the *pre-miR-27a* polymorphism and RCC risk

Allele frequencies and genotype distributions of *pre-miR-27a* rs895819 polymorphism in patients and controls are shown in Table 2. The observed genotype frequencies in the controls were consistent with that expected from HWE model (χ^2^ = 0.795, *P* = 0.373). The frequencies distribution of G allele significantly differentiated from A allele among cases and controls (*P* = 0.019). After adjusting for possible confounding factors (age, sex, smoking status, drinking status, hypertension, and diabetes), logistic regression analysis revealed that when comparing with AA homozygote, AG heterozygote was associated with a significantly reduced RCC risk (adjusted OR = 0.68, 95% CI = 0.53–0.87) and individuals with AG/GG genotype also had a reduced susceptibility to RCC (adjusted OR = 0.71, 95% CI = 0.56–0.90).

**Table 2:** Genotype and allele frequencies of rs895819 polymorphism among cases and controls and the associations with RCC risk.

Genotypes	Cases		Controls		Crude OR (95% CI)	Adjusted OR (95% CI)^a^	*P* b
	n	%	n	%			
AA	334	56.2	288	48.0	1.00 (reference)	1.00 (reference)	
AG	213	35.9	262	43.7	0.70 (0.55–0.89)	0.68 (0.53–0.87)	0.002
GG	47	7.9	50	8.3	0.81 (0.53–1.24)	0.78 (0.51–1.21)	0.274
AG/GG	260	43.8	312	52.0	0.72 (0.57–0.90)	0.71 (0.56–0.90)	0.004
G allele	307	25.8	362	30.2	0.81 (0.67–0.97)		0.019
*P_trend_*						0.020	

a Adjusted for age, sex, smoking, drinking status, diabetes and hypertension in logistic regression model.

b Two-sided χ^2^ test for either genotype distribution or allele frequency.

Furthermore, in the stratified analysis by age, BMI, sex, smoking status, drinking status, hypertension and diabetes, we found that the reduced risk was more pronounced in young subjects (adjusted OR = 0.56, 95% CI = 0.40–0.78), subjects with BMI≤24 (adjusted OR = 0.67, 95% CI = 0.48–0.93), males (adjusted OR = 0.66, 95% CI = 0.49–0.88), non-smokers (adjusted OR = 0.71, 95% CI = 0.53–0.95), non-drinkers (adjusted OR = 0.72, 95% CI = 0.55–0.95), subjects without hypertension (adjusted OR = 0.61, 95% CI = 0.46–0.81) and subjects without diabetes (adjusted OR = 0.69, 95% CI = 0.54–0.88) (Table 3).

**Table 3:** Stratification analyses between the genotypes of rs895819 polymorphism and RCC risk.

		Genotypes (cases/controls)						
Variables	Cases/controls	AA		AG/GG		AG/GG versus AA	AG/GG versus AA	*P* a
		n	%	n	%	Crude OR (95%CI)	Adjusted OR (95%CI)^a^	
Total	594/600	334/288	56.2/48.0	260/312	43.8/52.0			
Age (years)								
≤57	305/300	164/118	53.8/39.3	141/182	46.2/60.7	0.56 (0.40–0.77)	0.56 (0.40–0.78)	0.001
>57	289/300	170/170	58.8/56.7	119/130	41.2/43.3	0.92 (0.66–1.27)	0.93 (0.67–1.30)	0.670
BMI								
<24	292/326	160/148	54.8/45.4	132/178	45.2/54.6	0.69 (0.50–0.94)	0.67 (0.48–0.93)	0.016
≥24	302/274	174/140	57.6/51.1	128/134	42.4/48.9	0.77 (0.55–1.07)	0.76 (0.54–1.06)	0.104
Sex								
Male	378/402	211/188	55.8/46.8	167/214	44.2/53.2	0.70 (0.52–0.92)	0.66 (0.49–0.88)	0.005
Female	216/198	123/100	56.9/50.5	93/98	43.1/49.5	0.77 (0.52–1.14)	0.80 (0.54–1.18)	0.258
Smoking status								
Never	375/404	211/195	56.3/48.3	164/209	43.7/51.7	0.73 (0.55–0.96)	0.71 (0.53–0.95)	0.023
Ever	219/196	123/93	56.2/47.5	96/103	43.8/52.6	0.71 (0.48–1.04)	0.69 (0.46–1.03)	0.068
Drinking status								
Never	427/437	238/209	55.7/47.8	189/228	44.3/52.2	0.73 (0.56–0.95)	0.72 (0.55–0.95)	0.019
Ever	167/163	96/79	57.5/48.5	71/84	42.5/51.5	0.70 (0.45–1.07)	0.67 (0.42–1.05)	0.079
Hypertension								
No	378/433	212/191	56.1/44.1	166/242	43.9/55.9	0.62 (0.47–0.82)	0.61 (0.46–0.81)	0.001
Yes	216/167	122/97	56.5/58.1	94/70	43.5/41.9	1.07 (0.71–1.61)	1.00 (0.66–1.52)	0.997
Diabetes								
No	514/565	292/268	56.8/47.4	222/297	43.2/52.6	0.69 (0.54–0.87)	0.69 (0.54–0.88)	0.003
Yes	80/35	42/20	52.5/57.1	38/15	47.5/42.9	1.21 (0.54–2.69)	0.82 (0.32–2.07)	0.673

a Adjusted for age, sex, smoking, drinking status, diabetes and hypertension in logistic regression model. CI, confidence interval; OR, odds ratio.

### Association between *pre-miR-27a* polymorphism and clinical characteristics of RCC

 In addition, the association between *pre-miR-27a* rs895819 polymorphism and the clinical characteristics of RCC was also examined. Results showed that AG/GG genotype was associated with reduced susceptibility in localized clinical stage (adjusted OR = 0.71, 95% CI = 0.55–0.91) and similar effects were observed in well differentiated and poorly differentiated RCC (adjusted OR = 0.71, 95% CI = 0.55–0.93 for well differentiated, adjusted OR = 0.51, 95% CI = 0.28–0.93 for poorly differentiated) (Table 4).

**Table 4:** Associations between rs895819 polymorphism and clinical characteristics of RCC.

Variables	AG/GG		AA		AG/GG versus AA	AG/GG versus AA	*P* a
	n	%	n	%	Crude OR (95%CI)	Adjusted OR (95%CI)^a^	
Controls (n = 600)	312	52.0	288	48.0	1.00 (reference)	1.00 (reference)	
Cases (n = 594)							
Stage							
Localized (I+II)	213	43.8	273	56.2	0.72 (0.57–0.92)	0.71 (0.55–0.91)	0.006
Advanced (III+IV)	47	43.5	61	56.5	0.73 (0.47–1.11)	0.73 (0.47–1.11)	0.142
Grade							
Well differentiated (I+II)	173	44.0	220	56.0	0.73 (0.56–0.94)	0.71 (0.55–0.93)	0.010
Moderately differentiated (III)	68	46.3	79	53.7	0.80 (0.55–1.14)	0.80 (0.55–1.16)	0.235
Poorly differentiated (IV)	19	35.2	35	64.8	0.50 (0.28–0.90)	0.51 (0.28–0.93)	0.029

a Adjusted for age, sex, smoking, drinking status, diabetes and hypertension in logistic regression model.

### Interaction analyses of rs895819 polymorphism and risk factors

We have evaluated whether there were existence of interactions between rs895819 polymorphism and age, BMI, sex, smoking status, drinking status, hypertension and diabetes. As shown in Table S2, compared with individuals who were both ≤57 years and AA carriers, significantly reduced risk was observed in those who were ≤57 years with AG/GG genotype (adjusted OR = 0.57, 95% CI = 0.41–0.79) and those who were >57 with both AA and AG/GG genotypes (adjusted OR = 0.64, 95% CI = 0.46–0.89 for AA, adjusted OR = 0.60, 95% CI = 0.42–0.85 for AG/GG). Similar effects were found in AG/GG carriers which were <24 kg/m^2^, males, nonsmokers, nondrinkers, subjects without hypertension and subjects without diabetes when compared with AA carriers. We further observed rs895819 had multiplicative interactions with age and hypertension (*P*_interaction_ = 0.035 for age and *P*_interaction_ = 0.030 for hypertension).

In addition, Kaplan-Meier curves and log-rank tests were used to assess the association between the genotypes and survival of RCC. However, we did not find the rs895819 genotypes to be associated with the patients' overall survival. In the stepwise Cox regression, the results suggested that clinical stage was the best prognostic factor for RCC survival followed by tumor grade (data not shown).

### Association of rs895819 polymorphism with expression levels of hsa-miR-27a

We have collected 25 surgically removed normal renal tissue samples adjacent to tumors with different genotypes of rs895819, and the frequency distribution of the AA, AG, GG genotypes was 13, 10, and 2, respectively. We further assessed the association between the polymorphism and hsa-miR-27a expression levels using quantitative real-time RT-PCR. The samples with GG genotype was added to the samples of AG genotype for analysis. However, due to small samples of GG genotypes, we did not find any association between rs895819 and the expression of hsa-miR-27a (*P* = 0.08) (data not shown). Further functional studies were warranted when we collected large samples.

## Discussion

In the present study, we investigated the association between the *pre-miR-27a* rs895819 polymorphism and RCC risk. Considering relatively scarce frequencies of GG genotype, and similar scales of the ORs for heterozygote (AG) and homozygote (GG) the equality of that tested to observe a nonsignificant *P*-value of 0.568 suggesting a dominant model, we combined AG and GG genotypes as a dominant genetic model in the following association analysis. We found that individuals with AG/GG genotypes were associated with a significantly reduced RCC risk, compared with those carrying AA genotype. In addition, the effect was even stronger in non-smokers, young subjects, males and individuals without hypertension or diabetes. Besides, the subjects carrying G allele also had a reduced susceptibility to localized clinical stage, well differentiated and poorly differentiated RCC. To our knowledge, this is the first study to investigate the role of the *pre-miR-27a* rs895819 polymorphism in the risk of RCC.

Nowadays, increasing studies have suggested that miRNAs, which play an important role in cancer progress as tumor oncogenes or tumor suppressors are involved in crucial biological processes, including development, differentiation, apoptosis and proliferation [12], [21]. Genetic variations in miRNAs have been reported to be related with many tumors, such as breast cancer [22], gastric cancer [23], colorectal cancer [24] and lung cancer [15]. Though SNPs in miRNAs have been widely studied, the association between SNPs in miRNAs and renal cell cancer risk remains still unknown. Horikawa *et al.* defined 7 SNPs in 7 pre-miRNAs, and 10 SNPs in 8 pri-miRNAs, but none of them had a significant influence on RCC risk [25]. Nevertheless, Horikawa *et al.* suggested that genetic polymorphisms of the miRNA-machinery genes might affect RCC susceptibility individually and jointly.

SNP rs895819 was found in pre-miRNA regions of hsa-miR-27a, which was in chromosome 19, and it was located at position 40 relative to the first nucleotide. It has been reported that a variation causing structural change in the crucial region of miRNA could affect the maturation and the process of miRNA [26], [27]. We used the RNA-fold program to predict the most stable secondary structural of *pre-miR-27a* with two different sequences, but the G variation of rs895819 affected neither the conformation nor the free energy of *pre-miR-27a* (data not shown). In a word, the processing and maturation of miRNA was more complex and subtle than predicted. Zeng *et al.* observed that Drosha selectively cleaves RNA hairpins bearing a large terminal loop. When the loop is shortened by mutation or deletions, the maturation process will be impaired [28]. According to the predicted structure of *pre-miR-27a*, rs895819 is located in the center of the terminal loop, the rs895819 A>G transition may reduce the size of the loop. Consequently the cleavage by Drosha would be blocked, and the maturation process is thereby inhibited. In conclusion, rs895819 might play an important role in the maturation of miR-27a. However, due to we only had 2 individuals with GG genotypes, we did not find any association between rs895819 and the expression of hsa-miR-27a, these findings need to be confirmed by further studies.

To our knowledge, many human miRNA transcripts have target sequences in the 3′-UTR to which miRNAs bind and exert posttranscriptional repression. Liu *et al.* found *miR-27a* functions as an oncogene in gastric adenocarcinoma by targeting prohibitin [29] and Chintharlapalli *et al.* suggested that oncogenic *miR-27a* was a target for anticancer agent in colon cancer cells [30]. Our results showed that the wild genotype AA of *pre*-*miR-27a* had higher frequency in cases than in controls, whereas individuals carrying variant G allele had a reduced RCC risk, indicating that *miR-27a* was more likely to be an oncogene, which was consistent with previous studies [29], [30]. Mertens-Talcott *et al.* reported that in breast cancer cells, transfection of antisense miR-27a lead to increased expression ZBTB10 and these responses were accompanied by decreased expression of *survivin*, Moreover, survivin is a structurally unique member of the inhibitor of apoptosis protein family that suppresses apoptosis and regulates cell division. Despite the redundancy of cell death pathways, survivin appears to be required for cancer cell viability, and interference with survivin expression/function has been associated with catastrophic defects of mitotic transition and induction of mitochondrial-induced cell death [31]. Over-expression of survivin mRNA and protein were detected in RCC cell lines but not in normal human kidney epithelial cell line [19]. Elevated expression of survivin was also observed in RCC tissues compared with adjacent normal tissues [19], [32]. RCC patients with high survivin levels had a significantly shorter overall survival time than those with low levels [33], [34]. In other words, decreased miR-27a levels might reduce the incidence of RCC through suppressing the expression of survivn indirectly. That was why we focus on the *pre-miR-27a* polymorphism in RCC patients.

In this study, there were more hypertension patients and diabetics among the cases than among the controls, indicating that there was potential association between the two factors and RCC. We also found that rs895819 had multiplicative interactions with hypertension. It has been reported that certain types of renal tumors and cancer treatment could cause hypertension [35], [36] and a history of diabetes has been linked to renal cell cancer risk in several cohort studies [37], but its role independent of those of obesity and hypertension has not been demonstrated conclusively. Besides, our results indicated that the individuals with G allele have a reduced RCC risk in non-smokers. Cigarette smoking is the most consistently established causal risk factor for RCC, accounting for approximately 20% of cases of RCC [38]. Compared to never smokers, risk increased about 50% in male and 20% in female smokers [39]. Furthermore, after stratification for RCC clinical stage, it appeared that rs895819 GG genotype had a decreased risk of RCC with localized clinical stage. Thus, it was plausible that the variation was involved in the lower stage RCCs. However, this hypothesis needs to be confirmed in further studies.

As to the association between genotypes and overall survival of RCC, there was no significant result. The reason for this discrepancy may be that the mortality rate of our survival analysis is low due to our short follow-up time and the biological mechanisms that underlie the incidence of RCC may be different from those underlying prognosis, and even if the *miR-27a* polymorphism is involved in the etiology of RCC, that does not necessarily mean that it affects the outcome.

When interpreting our results, several limitations should be concerned. Firstly, our case-control study was a hospital-based study and it could not provide a good representation of overall populations. Nevertheless, the G allele frequency in our controls was similar to that in the HapMap database. Besides, the genotype distributions of the polymorphism in our controls conformed to HWE. Thus, the selection bias was unlikely to be substantial. Secondly, our sample size was relatively small. However, we had 83% power to detect a minimal OR of 0.68 with an exposure frequency of 30% under the current sample size. Thirdly, we did not perform the gene-environment interaction analysis due to lack of the detailed information of environment factors, such as occupational exposures, diet, and physical activities. However, our study also had several strengths. Firstly, it was the first study to investigate the association between *pre-miR-27a* rs895819 polymorphism and susceptibility to RCC. Secondly, we matched cases and controls by age and sex, and we obtained the results by adjustment of age, sex, smoking, drinking status, hypertension, and diabetes, which could reduce the influence of confounding factors.

In conclusion, our results provided the first insight into the contribution of *pre-miR-27a* rs895819 polymorphism to the risk of RCC in Chinese population. Although the association appeared to be statistically significant in our population, these findings should be further validated by larger, preferably prospective studies. For polymorphisms often vary in different ethnic groups, additional studies are also needed to clarify the association of this polymorphism with RCC risk in diverse ethnic populations.

## Supporting Information

Table S1 **The sequences of the primers and probe used to genotype the rs895819 polymorphism.**
(DOC)

Table S2 **Interaction analyses of rs895819 polymorphism and risk factors.**
(DOC)
