# Supplementary material for: A Genetic Variant in pre-miR-27a Is Associated with a Reduced Renal Cell Cancer Risk in a Chinese Population
Source: PLoS One. 2012 Oct 30;7(10):e46566. doi: 10.1371/journal.pone.0046566 (PMC3484143; doi:10.1371/journal.pone.0046566)
Supplement: Table S1 — The sequences of the primers and probe used to genotype the rs895819 polymorphism. (DOC) [file pone.0046566.s001.doc]

**Table S1.**The sequences of the primers and probe used to genotype the rs895819 polymorphism

| Name | Sequence (5’-3’) |
| --- | --- |
| rs895819 T>C-F | CGGAACTTAGCCACTGTGAACAC |
| rs895819 T>C-R | CTGAGGAGCAGGGCTTAGCT |
| rs895819 T>C-C | FAM-ACTTGGCGTGGACC-MGB |
| rs895819 T>C-T | HEX-CTTGGTGTGGACCC-MGB |
