# Supplementary material for: A Genetic Variant in pre-miR-27a Is Associated with a Reduced Renal Cell Cancer Risk in a Chinese Population
Source: PLoS One. 2012 Oct 30;7(10):e46566. doi: 10.1371/journal.pone.0046566 (PMC3484143; doi:10.1371/journal.pone.0046566)
Supplement: Table S2 — Interaction analyses of rs895819 polymorphism and risk factors. (DOC) [file pone.0046566.s002.doc]

**Table S2.** Interaction analyses of rs895819 polymorphism and risk factors

| Variables | Genotypes | Cases/Controls | | Adjusted OR (95%CI) ^a^ | *P^a^* |
| --- | --- | --- | --- | --- | --- |
|  |  | n | % |  |  |
| Age (years) | | | | | |
| ≤ 57 | AA | 164/118 | 53.8/39.3 | 1.00 (reference) |  |
| ≤ 57 | AG/GG | 141/182 | 46.2/60.7 | 0.57 (0.41-0.79) | <0.001 |
| > 57 | AA | 170/170 | 58.8/56.7 | 0.64 (0.46-0.89) | 0.008 |
| > 57 | AG/GG | 119/130 | 41.2/43.3 | 0.60 (0.42-0.85) | 0.004 |
| *P*_interaction_ (multiplicative) |  |  |  |  | 0.035 |
| BMI (kg/m^2^) | | | | | |
| < 24 | AA | 160/148 | 54.8/45.4 | 1.00 (reference) |  |
| < 24 | AG/GG | 132/178 | 45.2/54.6 | 0.67 (0.48-0.92) | 0.014 |
| ≥ 24 | AA | 174/140 | 57.6/51.1 | 1.07 (0.77-1.48) | 0.685 |
| ≥ 24 | AG/GG | 128/134 | 42.4/48.9 | 0.82(0.58-1.14) | 0.237 |
| *P*_interaction_ (multiplicative) |  |  |  |  | 0.626 |
| Sex | | | | | |
| Male | AA | 211/188 | 55.8/46.8 | 1.00 (reference) |  |
| Male | AG/GG | 167/214 | 44.2/53.2 | 0.67 (0.50-0.90) | 0.007 |
| Female | AA | 123/100 | 56.9/50.5 | 1.19 (0.82-1.72) | 0.365 |
| Female | AG/GG | 93/98 | 43.1/49.5 | 0.93 (0.63-1.37) | 0.723 |
| *P*_interaction_ (multiplicative) |  |  |  |  | 0.671 |
| Smoking status | | | | | |
| Never | AA | 211/195 | 56.3/48.3 | 1.00 (reference) |  |
| Never | AG/GG | 164/209 | 43.7/51.7 | 0.72 (0.54-0.96) | 0.025 |
| Ever | AA | 123/93 | 56.2/47.5 | 1.48 (1.00-2-21) | 0.053 |
| Ever | AG/GG | 96/103 | 43.8/52.6 | 1.01 (0.68-1.51) | 0.949 |
| *P*_interaction_ (multiplicative) |  |  |  |  | 0.907 |
| Drinking status |  |  |  |  |  |
| Never | AA | 238/209 | 55.7/47.8 | 1.00 (reference) |  |
| Never | AG/GG | 189/228 | 44.3/52.2 | 0.72 (0.55-0.94) | 0.018 |
| Ever | AA | 96/79 | 57.5/48.5 | 1.02 (0.68-1.55) | 0.909 |
| Ever | AG/GG | 71/84 | 42.5/51.5 | 0.70 (0.46-1.07) | 0.100 |
| *P*_interaction_ (multiplicative) |  |  |  |  | 0.861 |
| Hypertension |  |  |  |  |  |
| No | AA | 212/191 | 56.1/44.1 | 1.00 (reference) |  |
| No | AG/GG | 166/242 | 43.9/55.9 | 0.61 (0.46-0.81) | 0.001 |
| Yes | AA | 122/97 | 56.5/58.1 | 1.26 (0.88-1.80) | 0.202 |
| Yes | AG/GG | 94/70 | 43.5/41.9 | 1.23 (0.84-1.81) | 0.281 |
| *P*_interaction_ (multiplicative) |  |  |  |  | 0.030 |
| Diabetes |  |  |  |  |  |
| No | AA | 292/268 | 56.8/47.4 | 1.00 (reference) |  |
| No | AG/GG | 222/297 | 43.2/52.6 | 0.68 (0.53-0.86) | 0.002 |
| Yes | AA | 42/20 | 52.5/57.1 | 1.90 (1.08-3.34) | 0.026 |
| Yes | AG/GG | 38/15 | 47.5/42.9 | 2.28 (1.22-4.26) | 0.010 |
| *P*_interaction_ (multiplicative) |  |  |  |  | 0.186 |

^a^ Adjusted for age, sex, smoking, drinking status, diabetes and hypertension in logistic regression model.
